# Supplementary material for: Hermetic hydrovoltaic cell sustained by internal water circulation
Source: Nat Commun. 2024 Nov 12;15:9796. doi: 10.1038/s41467-024-54216-y (PMC11557918; doi:10.1038/s41467-024-54216-y)
Supplement: Supplementary file 1 — Supplementary Information [file 41467_2024_54216_MOESM1_ESM.pdf]

# Supplementary Information for

## Hermetic hydrovoltaic cell sustained by internal water circulation

Renxuan Yuan[1,2†], Huizeng Li[1†*], Zhipeng Zhao[1,2†], An Li[1], Luanluan Xue[1,2], Kaixuan Li[1], Xiao Deng[1,2], Xinye Yu[1,2], Rujun Li[1,2], Quan Liu[1,2], and Yanlin Song[1,2*]

[1]Key Laboratory of Green Printing, CAS Research/Education Center for Excellence in Molecular Sciences, Beijing National Laboratory for Molecular Science, Institute of Chemistry, Chinese Academy of Sciences, Beijing 100190, China.

[2]University of Chinese Academy of Sciences, Beijing 100049, China.

*Corresponding author. Email: lihz@iccas.ac.cn; ylsong@iccas.ac.cn.

**This PDF file includes:**

Supplementary Figures. 1 to 23
Supplementary Texts 1-6
Supplementary Table 1-4
Caption for video
References

**Other Supplementary Information for this manuscript include the following:**

Supplementary Video 1

## 1 Supplementary Figure

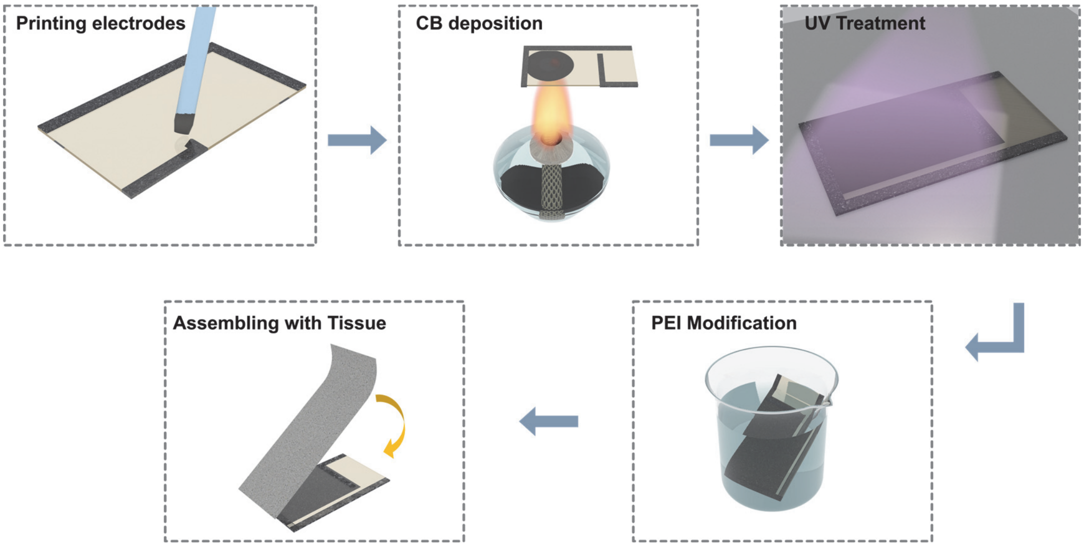


**Figure S1. Schematic diagrams of the HHC fabricating process.**

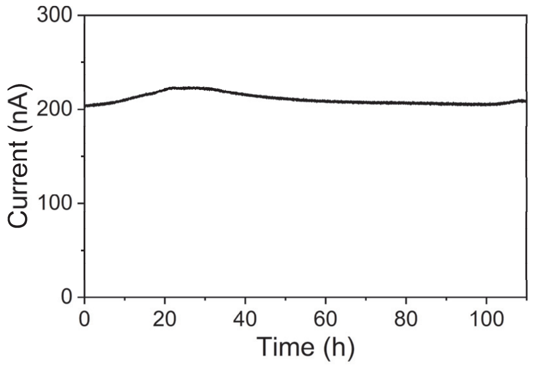


**Figure S2. The long-term short-circuit current performance of the HHC.** Source data are provided as a Source Data file.

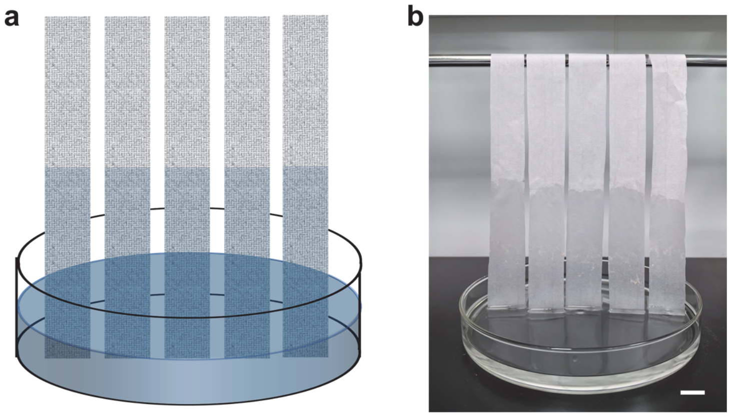


**Figure S3. The vigorous wicking phenomenon in tissue. a**, The schematic diagram of the experiment that evaluates the capillary wicking in the tissue. **b**, The photograph of the experiment. The tissue shows strong capillary effect with an average capillary height of $10.2 \pm 0.8$ cm, which should be more notable in hermetic cells. Therefore, the heterogeneous wicking bilayer structure is designed to adjust the capillary behavior for hydrovoltaic effect. Scale bar: 2 cm.

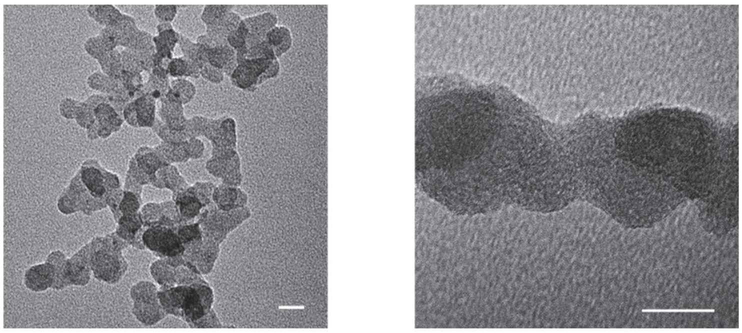

**Figure S4. Transmission electron telescope (TEM) images of the PEI modified CB particles at different magnifications.** The images show the diameters of the carbon black nanoparticles of 20-30 nm. Scale bars: 20 nm.

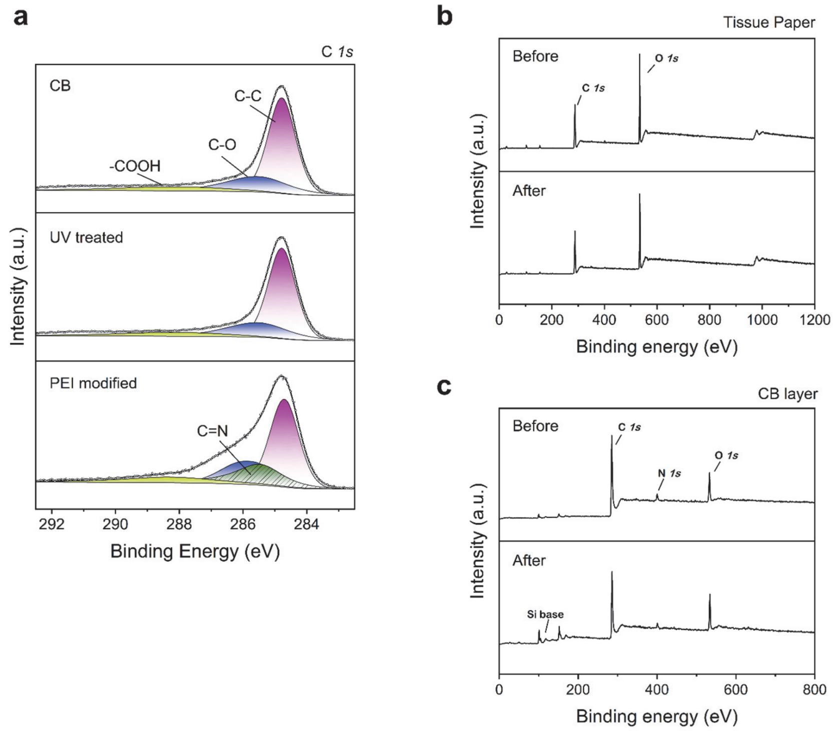


**Figure S5. The X-ray photoelectron spectroscopy (XPS). a,** The C 1s binding energy in the PEI modification process of the CB layer. **b** and **c,** The XPS full spectra analysis of **a,** the tissue paper layer, and **b,** the modified CB layer before and after the electricity generation process. Source data are provided as a Source Data file.

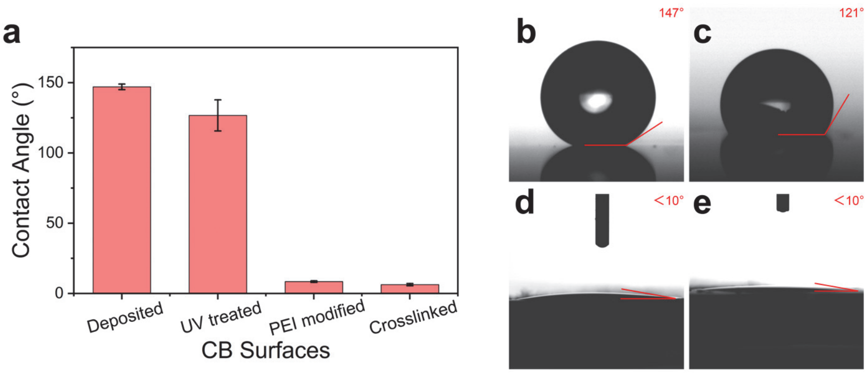


**Figure S6. The hydrophilicity enhancement of the CB layer after PEI modification. a**, The contact angles of the layer during the fabrication process. **b**-**e**, Optical graphs of 2 μL water droplets on different surfaces. **b**, The deposited CB layer was nearly superhydrophobic with a contact angle of 147 ± 1.8°. **c**, After ultraviolet treatment, the layer became less hydrophobic with a contact angle of 121 ± 9.9°. **d**, The PEI modified CB layer showed superhydrophilicity with a contact angle less than 10°. **e**, After crosslinked with glutaraldehyde solution, the CB layer became more reliable with an approximate contact angle of less than 10°. Data are collected from different devices (n=3, error bars represent SD). Source data are provided as a Source Data file.

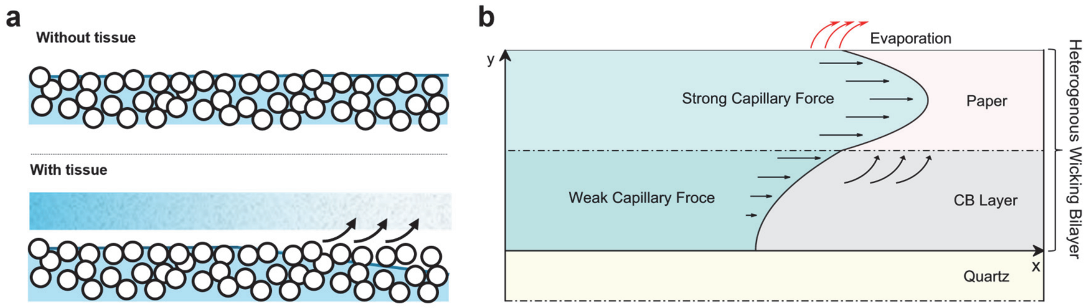


**Figure S7. Schematic diagrams to show the mechanism of the water distribution in the electricity generation units.** The tissue paper helps the bilayer to form the water content gradient, unlike the modified CB layer without tissue. Evaporation occurs on the outside surface of the tissue, maintaining a specific water distribution.

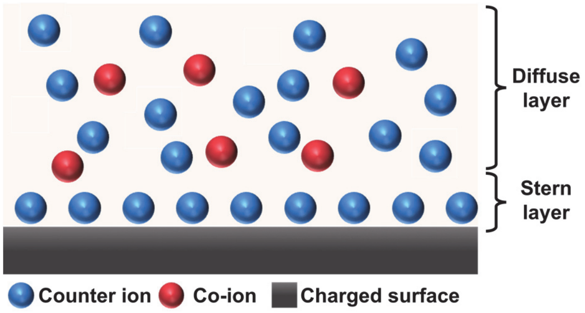


**Figure S8. The schematic diagrams the electric double layer (EDL).** The Stern layer is the region adjacent to the charged surface where counter ions in solution adhere by Coulomb interactions. Its thickness depends on the diameter of the counter ions. Next to the Stern layer is the diffuse layer, in which more co-ions are attracted.

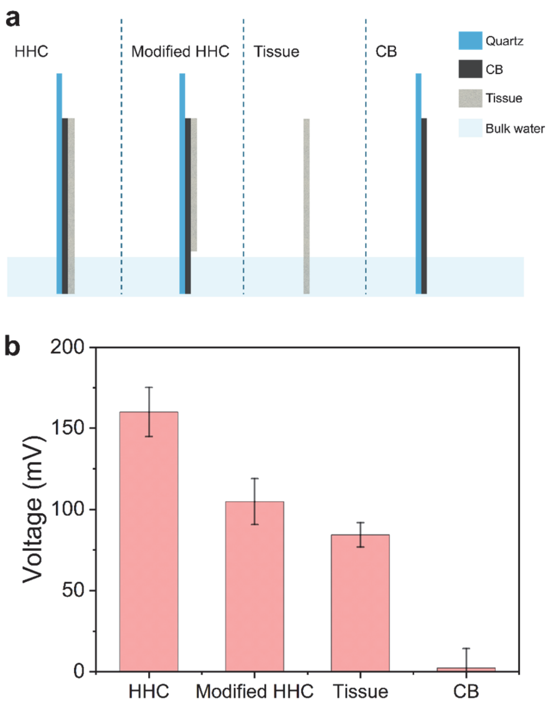


**Figure S9. The HHC with different design. a**, The diagram of the experimental design. The HHC, the HHC with tissue paper slight above the bulk water, the tissue paper, and the CB layer are tested. **b**, The open-circuit voltage of the HHC with different design. The adjustment of the tissue paper's position mainly affects the output performance by rebuilding the water content gradients in the CB layer and the tissue paper. Data are collected from different devices (n=3, error bars represent SD). Source data are provided as a Source Data file.

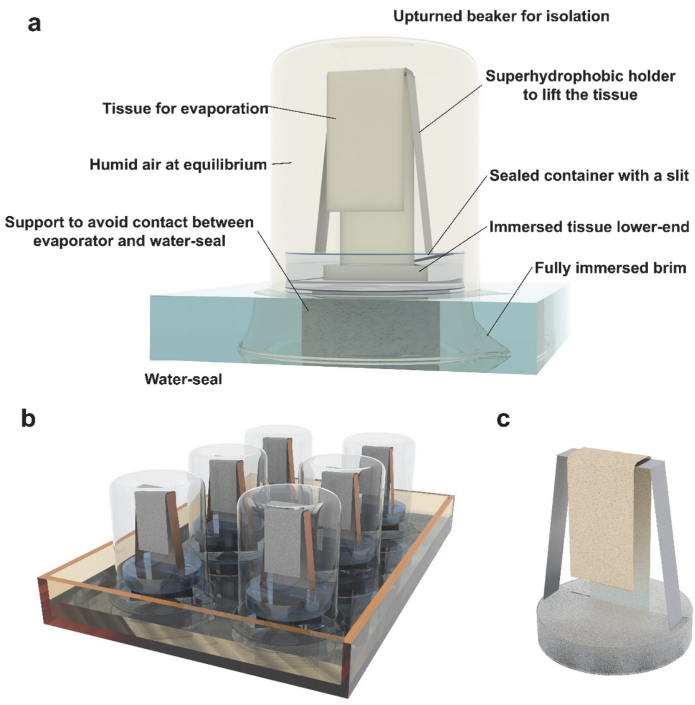


**Figure S10. The schematic diagram of the evaporation test.**

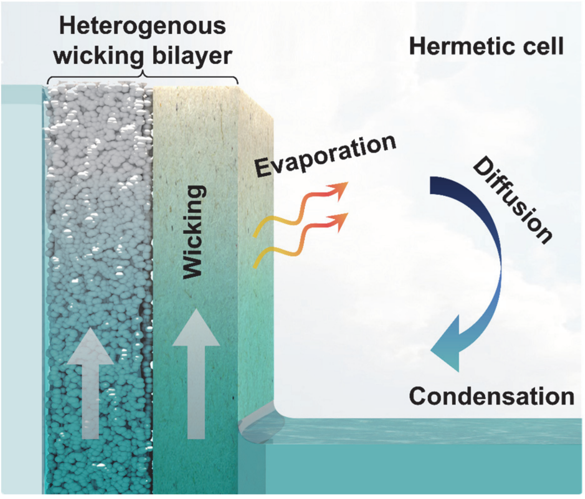


**Figure S11. Water transportation inside the hermetic cell to show the internal circulation process.**

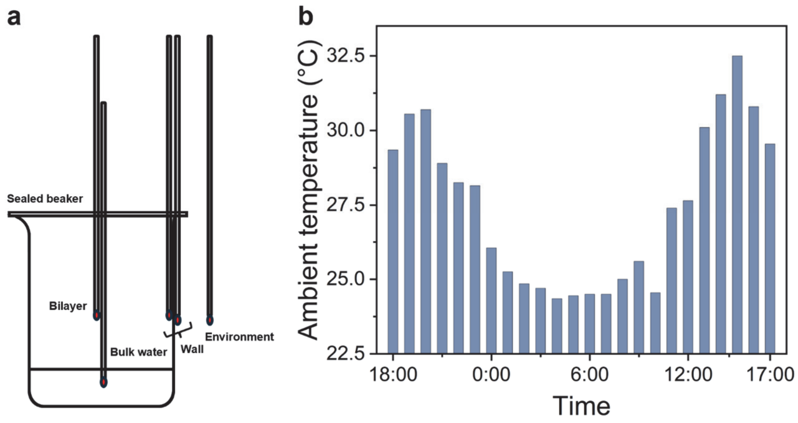


**Figure S12. a,** The diagram of the directly measurement of the temperature gradients in hermetic containers. **b,** The ambient temperature fluctuations with Fig. 2d. The temperature gradients at different locations in the sealed container are measured with fixed thermometers. The thermometers are calibrated in advance. Typically, the temperature of the inner wall of the container is the mean value of the values of both sides. Source data are provided as a Source Data file.

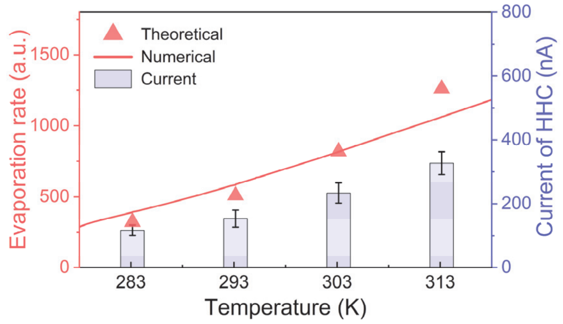


**Figure S13. The trends of evaporation rates with ambient temperature by theoretical method, numerical simulation, and experimental evaporation dependent currents.** The similar trends indicates that the theoretical analysis and the simulation by COMSOL are reasonable and dependable. Data of the currents are collected from different devices (n=3, error bars represent SD). Source data are provided as a Source Data file.

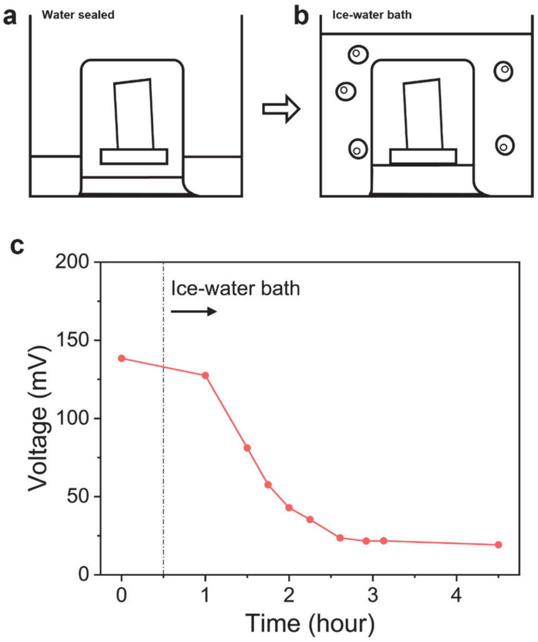


**Figure S14. The experiment design (a) and (b), and the open-circuit voltage of the HHC monitoring at nearly constant 0 °C.** In the first hour after placing to the ice-water bath, the temperature of the container wall decreases to 0 ℃, while the wet air inside the container remains warmer. Therefore, temperature gradient is formed between the bilayer regions and the wall. Water evaporates from the warmer electricity generation unit, and condensates on the colder wall of the container. Therefore, the voltage drops slowly in the first hour. When the temperature of the whole system approaches gradually to 0 ° C and remains stable, the temperature gradient inside the container, and also the electricity, minimizes. The final open-circuit voltage of 19 mV may result from the ineludible fluctuations of ice melting. Source data are provided as a Source Data file.

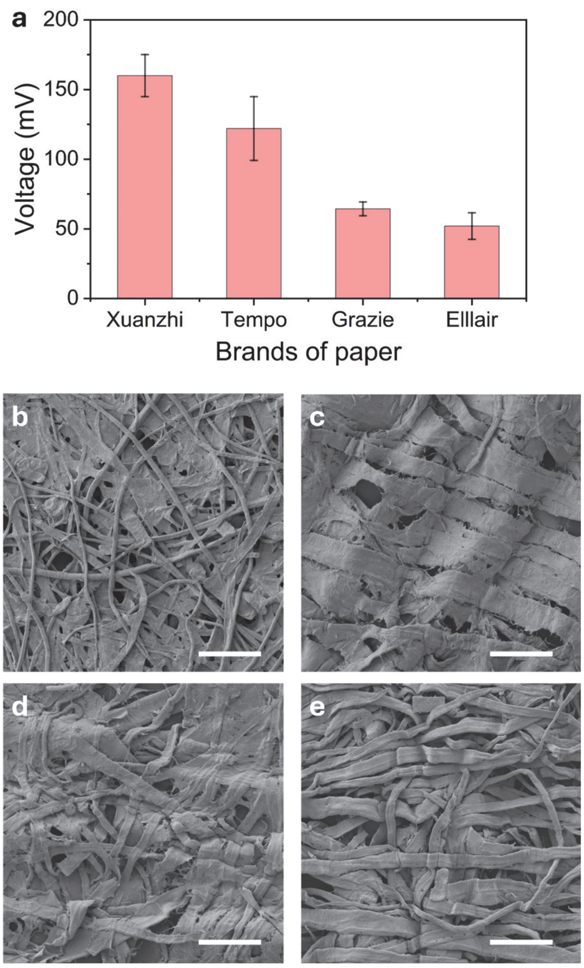


**Figure S15. The HHC with different kinds of tissue paper.** Xuanzhi, the Chinese traditional writing paper and three other commercial tissue papers (noted as their brands for short) are used in the HHC. **a**, The open-circuit voltage of the HHC with different papers. The scanning electron microscopy (SEM) characterization of **b**, Xuanzhi. **c**, Tempo. **d**, Grazie. **e**, Elleair. Scale bar: 100 um. The results show that the Xuanzhi with slender tubular fibers and complex networks generates larger output. Data are collected from different devices (n=3, error bars represent SD). Source data are provided as a Source Data file.

## 2 Supplementary Table

**Table S1. Data for the radar map in Fig. 1d.**

| | Normalized Efficiency | Stability | Working RH (%) | Voltage (mV) | Water consumption$^{-1}$ ($gh^{-1}$) |
|---|---|---|---|---|---|
| This work | 1 | 0.998 | 100 | 162 | 0 |
| Ref. 1 | 0.0352 | 0.75 | 70 | 1000 | 0.066 |
| Ref. 2 | 0.516 | 0.328 | 15 | 95 | 1.1 |
| Ref. 3 | 0.0135 | 0.972 | 50 | 353 | 0.69 |
| Ref. 4 | 0.504 | 0.707 | 45 | 550 | 0.066 |

The energy conversion efficiency represents the normalized power generated per unit of evaporation, which is mentioned in the main text and explained in Supplementary Text 3.5 in detail. The stability is the ratio of the voltage with the major dependent conditions to the voltage without these conditions, which are light and wind. The original collected data and calculation methods are provided in the Source Data file.

## 3 Supplementary Text

### 3.1 The mechanism of the electricity generation in the HHC

The hydrovoltaic electricity generation stems from the interaction between ionized solutions and charged surfaces. Based on the Gouy-Chapman-Stern theory, the electric double layer (EDL) is formed on the charged surfaces that contact with solution[5,6]. In the electricity generation unit, the hydrated hydroxide ions are adsorbed on/near the charged surface, while the positive co-ions distribute away from the charged surface. The Debye length, which indicates the influential range of EDL, can be classically described by Poisson-Boltzmann model:

$$\lambda_D = \left(\frac{\varepsilon_r \varepsilon_0 k_B T}{\sum_i \rho_{\infty i} e^2 z_i^2}\right)^{1/2} \tag{1}$$

where $\rho_{\infty i}$ is the number density of the ion types, $\varepsilon_r$ is the dielectric constant of the electrolyte, $\varepsilon_0$ is the permittivity of free space, T is the temperature, $k_B$ is the Boltzmann constant and $z_i$ is the ion valency. It clearly shows that the Debye length decreases with higher ion concentration. For our PEI/CB surface, the deionized water has a Debye length of several microns, while the aqueous solution of 0.1 M NaCl at 25 °C has a Debye length of 0.96 nm[5]. Interestingly, the size of the micro-nano channel in PEI/CB layer and the Debye length in the solution of low ion concentration are comparable. This similarity forms the overlapped EDL in the PEI/CB layer, which leads to a higher concentration of negative ions than positive ions in the channel, directionally moving with the capillary flow, shown in Fig. S7 and S8. Therefore, a streaming potential is generated by directional ion transports in the PEI/CB layer[1,7,8].

The electricity production in tissue layer can also be clarified by the Gouy-Chapman-Stern model with EDL. Regarded as a parallel-plate capacitor, the EDL has a potential drop that is related to the surface charges and solution properties to maintain the interfacial charge neutrality:

$$V_{EDL} = \sigma\left(\frac{d}{\varepsilon \varepsilon_0}\right) \tag{2}$$

where $\sigma$ is the surface charge density, $d$ is the thickness of the Stern layer, $\varepsilon$ is the dielectric constant of solution and $\varepsilon_0$ is the permittivity of free space[9]. The moisture content gradient in tissue leads to asymmetric adsorption of positive counterion, as a result of the equilibrium of capillary flow and evaporation. Moreover, the EDL on the tissue layer induces higher potential drop with higher MC, which causes potential difference between the wet and dry ends of the tissue by difference in surface charge density $\sigma$ (Fig. S16). Note that there is no current in the tissue layer due to its insulation. Hence a voltage is formed between the two ends of tissue based on the nonuniform ion adsorption. The positive ions migrate along with the capillary flow, which causes the change of the potential drop in the EDL. To this end, electrons on the CB surface would flow in the direction of positive ion migrations to maintain the charge neutrality, generating a current known as pseudo-streaming[10-13].

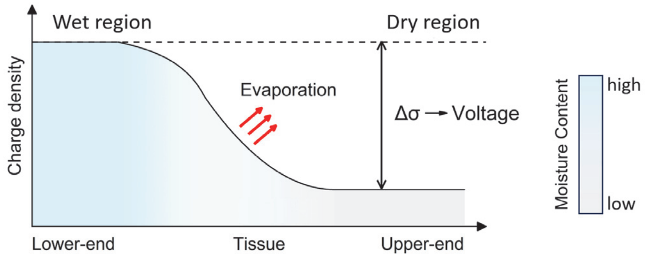


**Figure S16. Scheme to show the voltage generation from the tissue.** It is stemmed from the charge density difference between the lower and upper ends of the tissue.

### 3.2 The simulation of the water circulation by COMSOL

A model including a cylindrical container, a bulk water layer, an inside humid air domain, and a water domain at the bottom for water supply, is formed to study the water circulation in the HHC, as shown in Fig. S17. Both heat and mass transfers are simulated. The main processes happen as following. The outer space of air with a changing ambient temperature convects heat with the exterior walls of the container. The heat flows through heat conduction in the wall of the container and further the convection between the interior walls and the inner fluids (the air and the bulk water). The temperature gradient and the resulting water vapor pressure gradients are formed with the heat conduction and the diffusion of

water molecule in the air domain. Evaporation occurs on a single-sided water surface at the central area of the model, as well as the other wet surfaces. Condensation can happen on every exposed surfaces inside. Also, the heat transfer about the bulk water and the evaporation at the bottom of the container are simulated.

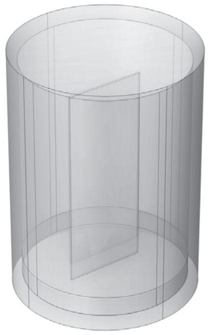

**Figure S17. The model geometry.**

The container is made of PMMA and contains water and air. Thin film water and quartz slice are employed to compose electricity generation unit at the central position. For modeling the water circulation, three effects must be considered: the heat transfer in all domains, the transport of moisture in nearly saturated air, as well as evaporation and condensation on surfaces.

For detailed conditions, three situations, including ambient temperature increasing, decreasing and fluctuating, are studied respectively with three processes. The ambient temperature changing rates are set as $\pm 1.2\ K/h$, based on measurements in Fig. 2e. Convection between the outside air of ambient temperature and the container occurs with a convection coefficient of $10\ W/m^2$ . Temperatures in all regions are the same with the environment at the beginning. Since the initial relative humidity of the humid air domain is set to 99.5 % for precondition, it takes several minutes to approach the steady states, resulting in the high net evaporating rates at the start of the simulation.

The processes of heat transfer, evaporation, moisture transport, as well as condensation are considered of significance in simulations to verify the water circulation. Hence, diagrams are employed to explain the complicated mechanisms. These indexes are found reasonable and stable during different temperature changes, which also meet the experimental measurements.

The driving temperature difference between the surface of the bilayer and the colder surfaces while both temperature rising and dropping are recorded after simulation of 600 mins (close to the diurnal cycle of temperature), shown in Fig. S18. The bulk water significantly changes its temperature with lower rates. Meanwhile, radial temperature distribution in the humid air is shown. Such temperature variations between different parts provide possible matter transfers from the vertical wet surface to the walls of the container and the bulk water surface.

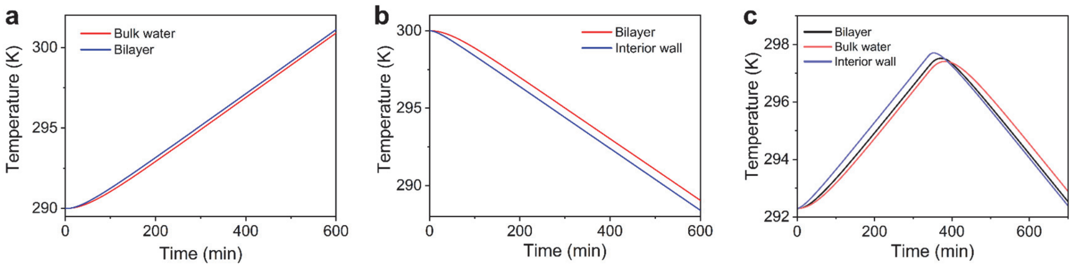


**Figure S18. The average temperatures on the evaporation and condensation surfaces. a,** The average temperatures of the surfaces of the bilayer structure and the bulk water with ambient temperature increasing by 1.2 $K/h$. **b,** The average temperatures of the surfaces of the bilayer structure and the interior wall with ambient temperature decreasing by 1.2 $K/h$. **c,** The average temperatures of the surfaces when the trend of temperature changes from increasing to decreasing. Source data are provided as a Source Data file.

Also, the comparison between the trend of the driving temperature differences and the simulated evaporation rates while temperature increasing and decreasing, are shown in Fig. 2g and Fig. 2h. The increasing periods at the first 100 minutes are the necessary time to reach nearly steady statuses, as mentioned above. The matched trends of the temperature differences and the evaporation rates verify the mechanism that the inner gradients of temperature and further the moisture concentration contribute to the water circulation.

Though it needs minutes to get to the equilibrium stage, the temperature differences are stably formed with a fixed ambient temperature change rate. The temperature difference turns out to be 0.2 K. Despite the disparity in the driving temperature difference during heating and cooling, several parameters, including the area of the condensation surfaces and the mass transport distance, vary from cases to cases, leading to similar evaporating

rates. On the other hand, based on the electricity generation mechanism in Supplementary Text 3.1, the open-circuit voltage measured in Fig. 1c is not obviously affected by the evaporation rates, while the short-circuit current is.

The moisture concentrations in the two simulations from the sectional view are demonstrated in Fig. S19. The moisture distributions are similar with the temperature distribution in Fig. 2g and Fig. 2h. Based on such concentration gradients, diffusion can take place in the container to form mass transports, which loops the water circulation.

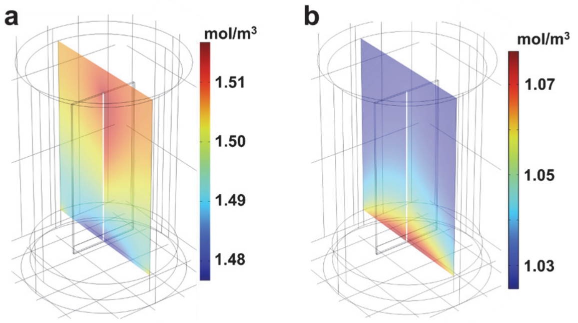


**Figure S19. The concentration distribution of water vapor from the sectional view while approaching the steady states.** The empty space at the center is the cross section of the bilayer structure. **a**, The concentration distribution with ambient temperature increasing by 1.2 K/h. **b**, The concentration distribution with ambient temperature decreasing by 1.2 K/h.

Heatmaps of net evaporation rates on the surface of the bilayer in two simulations are performed in Fig. S20. In these heatmaps, balanced and steady evaporation rates on the surface enable continuous wicking flow to generate electricity. Also, the evaporation on the edge of the tissue is found faster than that in the center, because the single-sided evaporation structure in the container provides larger diffusive region for the edge. Though the evaporation at different regions does not contribute to the power generation equally, causing difficulties in quantitative calculation of the power generation process, it is clear that the net evaporation can occur on the surface of the bilayer with the help by ambient temperature fluctuations.

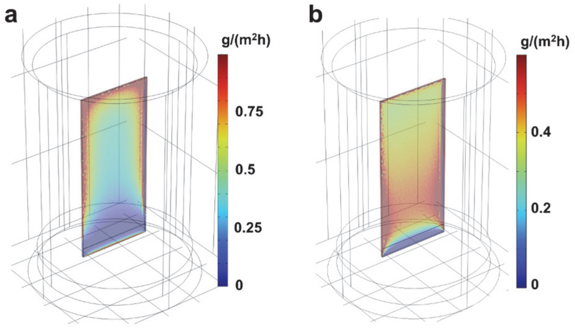


**Figure S20. The heatmap of the evaporation rate on the surfaces of the bilayer structure. a,** With ambient temperature increasing by 1.2 K/h. **b,** With decreasing by 1.2 K/h.

The 3D heatmaps of the moisture distributions and the moisture transport directions while temperature increasing and decreasing are plotted together to clear the tendency of the water transport in Fig. S21. The figure supports the mechanism in Supplementary Text 3.3. When the ambient temperature rise, the moisture tends to condensate on the surface of the bulk water with lower temperature. When the ambient temperature drops, the moisture tends to condensate on the surface of the interior wall of the container with lower temperature.

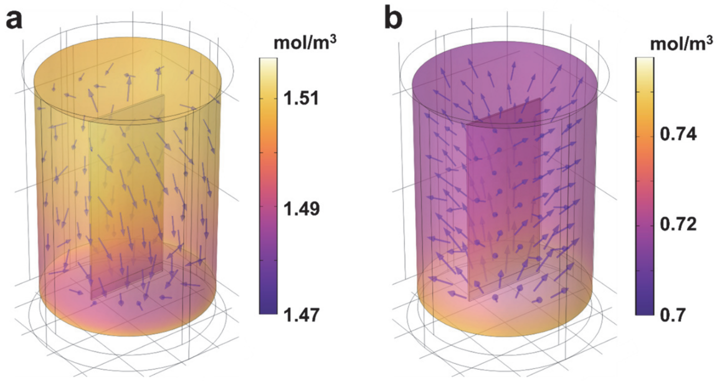


**Figure S21. The steady vapor concentration gradient in hermetic containers with ambient temperature changes. The arrows are the directions of the moisture transport. a,** Temperature increasing at 1.2 K/h in which the water molecules diffuse from the bilayer to the colder bulk water surface. **b,** Temperature decreasing at 1.2 K/h, in which the water molecules diffuse from the bilayer to the colder interior wall surface.

The average evaporation rates in the models can be recorded by time. To verify the relationship between the temperature gradient and the evaporation rate, Fig. 2i and Fig. j are plotted. The similar tendencies and the simultaneous drops suggest a positive correlation between the temperature difference and the evaporation rate that supports the mechanism in the coming Supplementary Text 3.3. The temperature gradient is the driving force of the water molecule transport from evaporation to condensation in the water circulation. Moreover, concerns on whether the model can rebuild the temperature and moisture gradients during fluctuations is solved. In Fig. S22, the situation that the trend of the ambient temperature changes from increasing to decreasing is simulated. The driving force of temperature difference changes from the difference between the bilayer and the bulk water, to the bilayer and the interior wall (Fig. S18c). The lines of surface temperatures intersect with each other, resulting in a drop of evaporation rate at the turning point. Meanwhile, the drop only reflects on a fluctuation in the short-circuit currents.

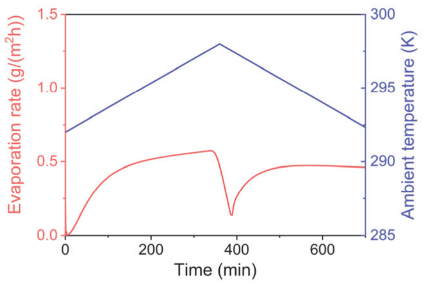


**Figure S22. The fluctuation of the average evaporation rate on the bilayer structure when the ambient temperature changes from increasing to decreasing.** Source data are provided as a Source Data file.

The modeling in COMSOL validates the water circulation process in the HHC. The cases with increasing, decreasing and fluctuating ambient temperature are proved with steady evaporation rates, and can be extended to the water circulation in the HHC.

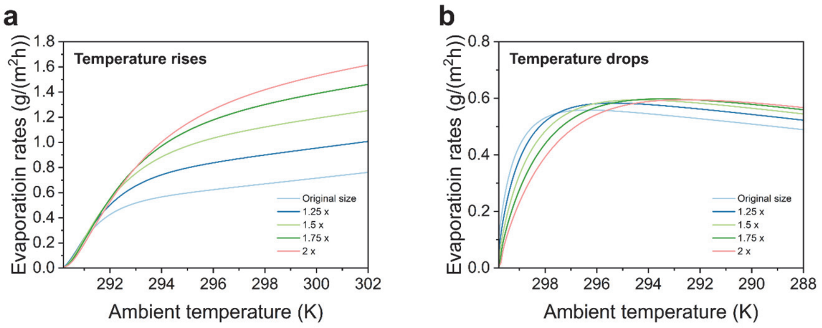


**Figure S23. The difference of the evaporation rates in different size of the containers while the ambient temperature (a) increasing and (b) decreasing.** Source data are provided as a Source Data file.

Fig. S23 shows the positive correlations between the size of the container and the evaporation rates. The cylindric container is scaled in all dimensions at the ratio of 1.25, 1.5, 1.75, and 2, varying in volumes from the original size to 8 times larger, with the same electricity generation unit. Also, when the ambient temperature increasing, the size of the container has greater influence than decreasing, because of the design of the HHC. Since the lower end of the electricity generation unit must be immersed in the bulk water, the distance of heat conduction and vapor transfer from the unit of the same area to the larger bulk water surface would not increase with larger containers. The bulk water influences the evaporation on the unit with its larger proportion in total water area. With larger container, the vapor from the unit condensates more on the bulk water surface while the ambient temperature increasing. As a competing process with the evaporation on the unit, net evaporation happens more on the bulk water surface in larger containers, while the ambient temperature decreasing. Therefore, the impacts of the size of the container differ while the temperature increasing and decreasing.

### 3.3 The mechanism of the evaporation in the HHC

The HHC harnesses ambient heat energy by hydrovoltaic effort with continuous evaporation inside a hermetic cell, which relies on local net evaporation in the HHC. Therefore, an explanation is needed to support that the HHC could generated stable output with steady water circulation in a hermetic container. Taking all involved processes into

consideration, we reveal that the temperature difference among the parts of the HHC caused by the diverse properties in materials and the continuous ambient temperature change, is the main factor for steady evaporation[14,15]. By the possible evaporation inside, the relative humidity is kept nearly saturated with frequent net evaporation and condensation. Due to the differences in heat conductivity and heat capacity, firm temperature difference is built during the ambient temperature changing process. For instance, because of the low heat conductivity of the air, slow heat transfer from the exterior walls of the container to the central part causes the radial temperature gradient in the container. Moreover, the high specific heat capacity of water leads to a hysteresis of the temperature change in the bulk water. Hence, when the ambient temperature increase, the temperature of the bilayer surface is higher than that of the bulk water surface. When the ambient temperature decreases, the temperature of the bilayer surface is higher than that of the interior wall of the container. Net evaporation happens on the hotter surfaces, always including the bilayer surface. Net condensation occurs on the colder surfaces, which is the bulk water in heating and the interior wall in cooling. The normal diffusion of water molecules from the bilayer to the colder surfaces dominates the water transport process from evaporation to condensation, which also limits the circulation rate inside the hermetic cell. We will further prove the rationality of the mechanism and deduce the analytical expression of the circulation rate, as well as the evaporation rate in this section.

**The heat transfer and the temperature distribution in the model**

In the HHC, during the whole working process, the ambient temperature fluctuation causes the temperature differences among the surfaces of different materials because of the differences in specific heat capacities and heat conductivities. The evaporation takes place on the wet surface of the wicking tissue, as the condensation occurs on the colder surface. The wicking tissue changes its temperature at a medium rate, which is supposed to be slower than the outer walls by its central position but quicker than the bulk water by the low specific heat of air. Therefore, when ambient heat goes up, the moisture transports to the colder water at the bottom based on a water molecule concentration gradient. On the other hand, the moisture transport to the colder walls of the container when the ambient temperature decreases. The temperature distribution in the HHC is described with classic equations to perform a moisture transport potential in the water circulation.

Firstly, a one-dimensional single-sided heated model is formed to match the heat transfer in humid air in the container. With high aspect ratio and low heat conductivity of air, the vertical heat transfer in the container can be simplified. The convective heat flux can be described by:

$$\dot{Q} = hA\Delta T \tag{3}$$

where $\dot{Q}$ is the heat transferred per unit time, $h$ is the convective heat coefficient, $A$ is the area, and $\Delta T$ is the between the fluid and the surface. Fourier's law states the relationship in thermal conduction:

$$q = -\lambda \frac{dT}{dx} \tag{4}$$

where $q$ is the local heat flux density, $\lambda$ is the heat conductivity, and $\frac{dT}{dx}$ is the temperature gradient. Supposed a steady temperature changing rate of ambient environment of $\frac{\partial T}{\partial t}$, with convection from ambient air to the exterior wall of the container and conduction in the walls, the temperature change of the interior wall ends up with a steady state that also changes by $\frac{\partial T}{\partial t}$. Set $T_w = c + \frac{\partial T}{\partial t} t$ as a precondition, where $T_w$ is the temperature of the interior wall, $c$ is a constant, and $t$ is the time. The temperature change of certain materials can be calculated by:

$$\Delta T = \frac{Q}{mC_p} \tag{5}$$

where $\Delta T$ is the temperature change, $Q$ is the heat change, $m$ is the mass of the material, and $C_p$ is the specific heat capacity. Let the spatial and temporal distribution in our one-dimensional model be $f(x,t)$, where $x$ is the distance. Supposed that a steady state of heat transport is approached after enough time, several boundary conditions can be inferred. The temperatures at certain distance changes at the same rate $\frac{\partial T}{\partial t}$, hence the relative temperature differences between any two points will not change by time. The temperature at one end equals to the temperature of the interior wall of the container $T_w$. And no heat transfer exists at the other end. According to the discussion above, we have the equations:

$$\begin{cases} f(x,t) + \frac{\partial T}{\partial t} dt = f(x, t+dt) \\ f(x_W, t') = c + \frac{\partial T}{\partial t} t \\ f'(0,t') = 0 \end{cases} \tag{6}$$

where $f'$ is the differential coefficient of $f$. From the equation (6), we have:

$$f'(x, t'+dt) - f(x,t')$$

$$= \frac{\frac{\big(f(x+dx,t') - f(x,t')\big) + \big(f(x-dx,t') - f(x,t')\big)}{dx} * \lambda * dt * A}{C_p * dx * A * \rho}$$

$$\frac{f'(x,t'+dt) - f(x,t')}{dt}$$

$$= \frac{\big(f(x+dx,t') - f(x,t')\big) + \big(f(x-dx,t') - f(x,t')\big)}{dx^2} \frac{\lambda}{C_p \rho}$$

$$\frac{\partial T}{\partial t} = \frac{\big(f(x+dx,t') - f(x,t')\big) + \big(f(x-dx,t') - f(x,t')\big)}{dx^2} \frac{\lambda}{C_p \rho}$$

$$= \frac{f'(x+dx,t') - f'(x,\ t')}{dx} \frac{\lambda}{C_p \rho} = f''(x,t) \frac{\lambda}{C_p \rho} \tag{7}$$

Therefore, we have:

$$f(x,t) = \frac{C_p \rho}{2k} \frac{\partial T}{\partial t} x^2 + c + \frac{\partial T}{\partial t} t, x \in [0, x_w] \tag{8}$$

for the temperature distribution in the air, where $x_w$ is the distance from central to the wall of the container. The temperature difference between both end at the ideal steady state is:

$$f(x_w, t) - f(0,t) = \frac{C_p \rho}{2k} \frac{\partial T}{\partial t} x_w^2 \tag{9}$$

Given the ideal steady state in one-dimensional model, the complex situation in two-dimensional model is studied. Since the multidirectional heat transfer cannot be neglected in the case of bulk water, a two-dimensional axisymmetric model is employed. Similarly, the temperature distribution in a two-dimensional model is set as $g(r,z,t)$, where $r$ and $z$ are the axes. According to the equations (4) and (5), we have:

$$\frac{g(r,z,t+dt)-g(r,z,t)}{dt}$$
$$=\frac{k}{c_p}\left(\frac{g(r+dr,z,t)+g(r-dr,z,t)-2g(r,z,t)}{dr^2}\right.$$
$$\left.+\frac{g(r,z+dz,t)+g(r,z-dz,t)-2g(r,z,t)}{dz^2}\right)$$

$$\frac{\lambda}{C_p}(g_r''+g_z'')=\frac{\partial T}{\partial t} \tag{10}$$

where $g_r''$ and $g_z''$ are the second derivative of $g(r,z,t)$. It is difficult to solve equations above, hence detailed calculations are performed with the numerical method in Supplementary Text 3.2.

Hence, we employ the models to further analysis the scale of the temperature differences caused by ambient temperature fluctuations. The radial temperature distribution in the air can be simplified as a one-dimensional heat transfer in equation (9), from which we learn the driving condition in the cooling process. The temperature difference in the heating process happens between the tissue surface and the surface of the bulk water. An average value of the surface temperature of the bulk water can be calculated for comparison with the temperature of the bilayer structure.

Several conditions, including the heat exchange between the water and the air, the heat capacity of the bilayer, are not taken into account, because of their small impact. Though it is difficult to consider the problem comprehensively from a practical situation with unpredictable ambient conditions and asymmetric structures, the temperature differences among the different parts in the hermetic container can be identified at the scale of $10^{-1}$ K with the analysis above. The temperature differences enable the continuous evaporation, moisture transport and the moisture gradient inside the container that contributes to the water circulation.

**The mass transfer by diffusion**

The diffusion process completes the mass transfer path from evaporation to condensation. Diffusion based on concentration gradients performs among the different surfaces in the model driven by the temperature gradients[16,17]. Fick's first law provides the relationship

between the diffusion flux and the gradient of the concentration:

$$J = -D\frac{d\varphi}{dx} \tag{11}$$

where $J$ is the diffusive flux, $D$ is the diffusion coefficient, and $\frac{d\varphi}{dx}$ is the concentration gradient. During the diffusion process, the local water vapor pressure is not necessarily lower than the local saturated vapor pressure, since the condensation of slightly oversaturated humid air needs condensation nucleus or walls. Hence, only the temperature difference between both ends is taken into consideration to analyze the concentration gradient. The relationship between the saturated vapor pressure and the local temperature is given by the Clausius-Clapeyron equation:

$$\frac{dp}{dT} = \frac{L}{T\Delta V} \tag{12}$$

where $L$ is the latent heat of evaporation, $T$ is the average temperature, $p$ is the vapor pressure at $T$. From the ideal gas law $pV = nRT$, where $n$ is the number of moles of the given gas, with a reasonable approximation, we have:

$$\Delta p = p\left(1 - \exp\left(-\frac{L\Delta T}{RT^2}\right)\right), when\ \Delta T \ll T. \tag{13}$$

where $\Delta p$ and $\Delta T$ are the changes in $p$ and $T$, respectively, and $R$ is the standard gas constant. From equation (12), we have:

$$\rho = \frac{Mn}{V} = \frac{Mp}{RT} \tag{14}$$

where $M$ is the molar mass of water. Furthermore, from equations (11) and (14), we have:

$$J_d = -D\frac{M\Delta p}{RT\Delta x} \tag{16}$$

which is the expression for one-dimensional diffusion.

**The expression of evaporation rates in the HHC**

The diffusion process between the surfaces with temperature difference limits the evaporation rate on the bilayer, with an overall consideration on interfacial transfers and wicking, proved in the coming parts. The diffusive flux can be detailed described by

combining equation (10), (12) and (16) of heat and mass transfer:

$$J_d \sim \frac{aDMp\left(1 - exp\left(-\frac{L\Delta T}{RT^2}\right)\right)}{RTx_w} \tag{17}$$

where a constant $a$ is employed to cover the inaccuracy of the one-sided evaporation and the cylinder structure in our practical 3D container. Based on experimental results on the evaporation rate (Fig. 2c), the evaporation rates while temperature increasing, decreasing and fluctuating are close to the practical value. We employ the minimal expression of temperature difference with increasing temperature to represent the evaporation rate in the HHC, since the evaporation rate is stable during the monitoring. To further simplify the equation (17), we neglect some constant relations and employ the Taylor expansion of $\exp(x) = 1 + x + \frac{x^2}{2!}$... in equation:

$$\dot{m} \sim J_d \sim \frac{DMpLC_{p,a}\rho_a x_w}{\lambda R^2 T^3}\frac{\partial T}{\partial t} \tag{18}$$

A conclusion is further employed:

$$\dot{m} \sim k\frac{DpL}{T^3}\frac{\partial T}{\partial t} \tag{19}$$

where $k = MC_{p,a}\rho_a x_w/\lambda R^2$ is used to summarize the constants in the expression. The diffusion flux while heating $J_d$, which is supposed to equal to the evaporation rate $\dot{m}$ on the bilayer structure with increasing temperature in the HHC, is calculated to be ~$10^{-1}$ g/($m^2$h), similarly to the experimental result. However, the evaporation rates have positive correlation with the ambient temperature as the exponent apparently indicates. The saturated vapor pressure $p$, the diffusive coefficient $D$, the specific heat capacity of air $C_{p,a}$, and the density $\rho$ are related to the ambient temperature. The evaporation rate indeed shows positive correlation with temperature, shown in Supplementary Text Table. 1. Such relationship also reflects in measurements that the short-circuit current increases with higher temperature.

**Table S2. The values of the parameters and the evaporation rates at different temperatures in equation (18), which are employed in Fig. S13.**

| $T/℃$ | 10 | 20 | 30 | 40 |
|---|---|---|---|---|
| $D/(10^{-5}m\cdot s^{-1})$ | 2.3 | 2.42 | 2.55 | 2.68 |
| $p/Pa$ | 1312 | 2339 | 4246 | 7381 |
| $L/(kJ\cdot mol^{-1})$ | 44.63 | 44.2 | 43.78 | 43.35 |
| $C_{p,a}/(J\cdot kg^{-1}\cdot K^{-1})$ | 1005 | 1006 | 1006 | 1007 |
| $\rho_{ha}/(kg\cdot m^{-3})$ | 1.25 | 1.2 | 1.16 | 1.13 |
| $\lambda_a/(mW\cdot m^{-1}\cdot K^{-1})$ | 25.1 | 25.9 | 26.6 | 27.4 |
| $(\dot{m}/a)/(g\cdot m^{-2}\cdot h)$ | 0.046 | 0.073 | 0.117 | 0.180 |

**The influence of other factors**

The evaporation and the condensation on the bilayer structure caused by temperature fluctuation induced changes of saturated vapor pressure, are taken into consideration. Supposed that all changes on vapor concentration reflect on interfacial mass transfer, based on equation (13) and (14), a mass concentration changing rate of water vapor can be calculated turning out to be ~$10^1$ g/($m^3$h). Given the distribution of condensation area and the boundary temperatures, the condensation rate on the bilayer is supposed to be $10^{-3}g/(m^2\cdot h)$, which is significantly much less than the net evaporation rate $E$ above. Therefore, the influence of changes of saturated vapor pressure is ignored during the temperature fluctuation. The calculation also explains the similar performances of the HHC while temperature increasing and decreasing processes.

The "rate determining step" in the water circulation can be proved as diffusion with moisture gradient. Other processes might get involved including evaporation, condensation and wicking. Regardless of the fast-wicking process, the Hertz-Knudsen equation describes the maximum rates of evaporation and condensation at an interface[18]:

$$\dot{m}_{max} = \left(\frac{M}{2\pi R}\right)^{\frac{1}{2}} \left(\sigma_e \frac{p_{eq}}{\sqrt{T_i}} - \sigma_c \frac{p_v}{\sqrt{T_v}}\right) \tag{20}$$

where $\dot{m}_{max}$ is the local evaporation flux determined by interfacial exchange, $M$ is the molecule weight, $R$ is the gas constant, $\sigma_e$ and $\sigma_c$ are the evaporation and condensation, $p_{eq}$ is the equilibrium vapor pressure of the liquid, $p_v$ is the partial vapor pressure in gas phase, and $T_i$ and $T_v$ are the temperatures for interface and vapor. The coefficients $\sigma_e$ and $\sigma_c$ are the empirical values varying from 0.01 to1, mostly regarded as equal[19]. Given the equation (13), we can infer an approximate value for the evaporation rate of $10^3$ g/(m$^2$h), which is significantly larger than the diffusion rate. Hence, the mass exchange rate at the interface is not limited by the molecular collisions[20].

The asymmetric structures, the single-sided evaporation surface, the vertical heat transfer in air, and the weak flows in the HHC are unachievable with detailed expressions, which may reduce the accuracy of the result. However, these aspects have little influence on the evaporation rate, which are taken into consideration in the modeling in Supplementary Text 3.2. Our analysis not only proves the feasibility of the internal water circulation in a hermetic container, but also qualitatively proposed the approximate solution of the evaporation rates and the impacts of the set conditions. Equation (19) provides available relationships of the parameters for the further improvements of HHC.

Also, a comparison among evaporation rates at different temperatures of the numerical values in Supplementary Text 3.2 and the theoretical values in this section, is summarized with the evaporation-depended currents of the HHC in Fig. S13. The match of the trends of the evaporation rates at different temperatures from different methods indicates the authenticity of the mechanism that claims the interior temperature difference as the major driver of the continuous evaporation and the water circulation inside the HHC. The parameters mentioned in this section are summarized in the Supplementary Text Table. 2 with values at 298.15 K.

**Table S3. Physical quantities, their values and units mentioned above.**

| Physical quantities | Abbreviations | Values | Units |
|---|---|---|---|
| Free convection coefficient of air | $h$ | 5~50 | $W/(m^2 \cdot K)$ |
| Heat conductivity of water | $k_w$ | 0.6 | $W/(m \cdot K)$ |
| Ambient temperature changing rate | $\frac{\partial T}{\partial t}$ | ±0.6~1.2 | $K/h$ |
| Specific heat capacity of water | $C_{p,w}$ | $4.2 \times 10^3$ | $J/(kg \cdot K)$ |
| Radius of the cylindrical container | $r_w$ | ~0.05 | $m$ |
| Depth of the bulk water | $z_w$ | ~0.01 | $m$ |
| Standard gas constant | $R$ | 8.314 | $J/(mol \cdot K)$ |
| Molar mass of water | $M$ | $1.8 \times 10^{-2}$ | $kg/mol$ |

### 3.4 The influential factors on the output performance

For the HHC, several factors can influence the voltage by affecting the surface charge density when DI water is used as circulation solution. For instance, the voltage goes up with rapid changes in temperature and intense light, as the excessive evaporation at the upper-end of the tissue leads to the decrease of the counter-ions adsorption. By comparison, the moisture content at the lower-end is sustained by the capillary wicking from a short distance. As a result, the surface charge density on the upper-end of the tissue reduces while the lower-end remains unchanged, thus enlarging the potential difference[21].

When salt solutions, such as NaCl aqueous solution, are used as the circulation solution, the lower-end of the tissue can adsorb more ions than that at the upper-end, thus enlarging the voltage generated by the tissue. On the other hand, the steaming potential generated by the PEI/CB layer lowers with the increase of the NaCl solution concentration[7], and is dominant to the output voltage.

Dissimilarly, the current of our HHC mostly depends on the evaporation rates inside the HHC. Since the current generation is accompanied by directional ion transport along with the capillary flow, the current increases with higher ionic mobility[10,15,22]. Environmental factors, such as temperature and light, accelerate the evaporation and promote the water circulation, thereby enlarging the current.

### 3.5 The efficiency of the HHC and the other reported hydrovoltaic generators

The electricity generation of hydrovoltaic devices depends on the water evaporation induced by ambient heat. Unlike other thermal engines that output power from high grade energy sources like petroleum and liquefied gas, it is difficult to calculate the input energy (including the ambient heat and the solar energy) for the hydrovoltaic devices, as well as the energy efficiency $\eta = {}^{W}/_{Q_{input}}$, where $\eta$ is the efficiency, $W$ is the output, and the $Q_{input}$ is the overall input energy. Therefore, recent research progresses generally uses the open-circuit voltage and/or short-circuit current as a measurement of the output performance.

Nevertheless, we come up with the method that only focus on the energy conversion process in evaporation phenomenon. That is, calculating the proportion of generated electricity in the total output energy. It is possible to analysis the most output energy, which is the heat of evaporation and the output electricity. Therefore, since the evaporation rates can be easily measured, we have an approximate formula for efficiency of hydrovoltaic generators:

$$\eta = \frac{W_e}{W_e + E_w + W_d} \approx \frac{VIt}{2Lm} \tag{21}$$

where $W_e$ is the work done by electricity, $E_w$ is the heat of vaporization, $W_d$ is the dissipation energy like heat, $V$ and $I$ are the open-circuit voltage and the short-circuit current of the hydrovoltaic generators, $t$ is the time, and $m$ is the total evaporation amount. Since most literatures records the evaporation rates of their devices, we might have the approximate efficiency to compare the ability among the devices.

Many representative hydrovoltaic devices are listed below with their performances,

including open-circuit voltages, short-circuit currents, evaporation rates, and the combining methods. Sometimes, the hydrovoltaic methods do not design special structures to achieve higher evaporation rates, which could be regarded as 0.22 kg/m$^2$h, the evaporation rate on clam water surface. With the reported data, we could calculate the approximate energy conversion efficiency for some devices, shown in Table S4. The formula for specific calculation is:

$$\eta \approx \frac{VI}{2L\dot{m}A}$$

where $\dot{m}$ is the evaporation rates, $A$ is the area of the devices.

Though the open-circuit voltages and the short-circuit currents of the devices differ by more than hundredfold, regardless of the devices with combining methods, taking the size of the devices into consideration, most devices in Table S4 have similar energy conversion efficiencies. Employing the same mechanism, in which the water molecules interact with the charged surface, these devices generate electricity from evaporation process with the energy conversion efficiency of a similar order of magnitude. Limited by its hermetic structure, unlike other devices in Table S4, the HHC holds an evaporation rate of ~10$^{-3}$ kg/(m$^2$h), which is 1000 times smaller than the other reports. Therefore, with the same mechanism and similar efficiencies, our HHC method inevitably generates lower outputs. Even though, the HHC performs better in other important aspects, including the range of applications and the water consumption.

**Table S4. The reported hydrovoltaic devices and their performances.**

| **Voltage (V)** | **Current (μA)** | **Evaporation rate (kg/(m$^2$h))** | **Combining methods** | **Efficiency (10$^{-4}$%)** | **Reference** |
|---|---|---|---|---|---|
| 0.4 | 14 | - | - | 15.6 | 23 |
| 0.28 | 55 | - | - | - | 24 |
| 1.2 | 0.49 | - | - | 1.96 | 25 |
| 0.3 | 100 | - | Galvanic effect | - | 12 |

| | | | | | |
|---|---|---|---|---|---|
| 0.084 | 500 | 1.15 | Galvanic effect | 2.68 | 26 |
| 0.25 | - | 0.2 | - | 0.102 | 27 |
| 0.24 | 42.7 | - | Moisture adsorption | 18.9 | 28 |
| 0.74 | - | - | Galvanic effect | 1.66 | 22 |
| 0.16 | 20 | - | Galvanic effect | - | 29 |
| 2.5 | 0.4 | - | - | 0.83 | 30 |
| 1 | 0.1 | - | - | 1.11 | 1 |
| 0.432 | 64.2 | 2.78 | - | 3.46 | 2 |
| 0.5 | 0.25 | - | - | - | 31 |
| 1.48 | 0.037 | - | Ethanol evaporation | - | 32 |
| 0.71 | 0.38 | 1.866 | Galvanic effect | 0.30 | 33 |
| 0.6 | 0.12 | - | - | 0.20 | 33 |
| 0.37 | - | 1.3 | - | 10.1 | 34 |
| 0.55 | 22 | 1.15 | - | - | 35 |
| 0.31 | 5.3 | 1.3 | - | 0.31 | 36 |
| 0.37 | - | 1.3 | - | - | 34 |
| 0.36 | 13 | 2.78 | - | 0.68 | 3 |
| 0.778 | 32 | 1.74 | Galvanic effect | | 4 |
| 0.022 | 144 | 2.26 | - | 0.16 | 37 |
| 0.73 | 0.6 | 2.38 | - | 0.045 | 38 |
| 0.16 | 0.2 | $10^{-3}$ | - | 31.3 | Our work |

The calculation of efficiency is also employed in Fig. 1d.

### 3.6 The output measurement of external resistances

The HHC is connected with a series of external resistances to test its output performance. The expressions for output of resistors are derived from the equivalent circuit, as the inset shown in Fig. 4a, where the HHC is considered as a DC power supply with an internal resistance. We have:

$$V_{OP} = \frac{R}{R+r} \times V_{OC} \tag{21}$$

$$I_{SC} = \frac{V_{OC}}{R+r} \tag{22}$$

$$P_{OP} = V_{OP} \times I_{SC} \tag{23}$$

where $V_{OP}$ is the output voltage across the external resistor, $R$ is the resistance of external resistor, $r$ is the internal resistance of the HHC, $V_{OC}$ is the open-circuit voltage of the HHC, $I_{SC}$ is the short-circuit current, $P_{OP}$ is the output power on the external resistor. By fitting the voltage data in Fig. 4a (the black dash curve), we find that the internal resistance of the HHC is fixed, regardless of the external resistance. Therefore, the HHC can be regarded as an ideal power resource. Based on this, the output power can be inferred by equation 5, with the maximum value of 8.24 nW at an external resistance of 0.7 MΩ.

## 4 Caption for Video

**Supplementary Video 1. Electronics powered up by HHC array.** A 24-HHC-array that is connected in series can power up commercial electronic devices with capacitors, including LCD screens, digital clocks, calculators and LEDs. A clock is used to display the actual time.

## Reference


1 Xue, G. *et al.* Water-evaporation-induced electricity with nanostructured carbon materials. *Nat. Nanotechnol.* **12**, 317-321 (2017).

2 Sun, Z. *et al.* Achieving efficient power generation by designing bioinspired and multi-layered interfacial evaporator. *Nat. Commun.* **13**, 5077 (2022).

3 Wan, Y. *et al.* Bird's nest-shaped $Sb_2WO_6$/D-Fru composite for multi-stage evaporator and tandem solar light-heat-electricity generators. *Small* **20**, e2302943 (2024).

4 Ma, J. *et al.* Achieving solar-thermal-electro integration evaporator nine-grid array with asymmetric strategy for simultaneous harvesting clean water and electricity. *Adv. Sci.* **10**, e2303815 (2023).

5 Schoch, R. B., Han, J. & Renaud, P. Transport phenomena in nanofluidics. *Rev. Mod. Phys.* **80**, 839-883 (2008).

6 Zhang, Z. *et al.* Emerging hydrovoltaic technology. *Nat. Nanotechnol.* **13**, 1109-1119 (2018).

7 Zhang, S. Y., Chu, W. C., Li, L. X. & Guo, W. L. Voltage distribution in porous carbon black films induced by water evaporation. *J. Phys. Chem. C* **125**, 8959-8964 (2021).

8 Persson, B. N. J., Tartaglino, U., Tosatti, E. & Ueba, H. Electronic friction and liquid-flow-induced voltage in nanotubes. *Phys. Rev. B* **69**, 235410 (2004).

9 Brown, M. A., Goel, A. & Abbas, Z. Effect of electrolyte concentration on the Stern layer thickness at a charged interface. *Angew. Chem. Int. Ed. Engl.* **55**, 3790-3794 (2016).

10 Yun, T. G., Bae, J., Rothschild, A. & Kim, I. D. Transpiration driven electrokinetic power generator. *ACS Nano* **13**, 12703-12709 (2019).

11 Yoon, H., Cheong, J. Y., Yun, T. G. & Hwang, B. Cellulose fiber-based, yarn-based, and textile-based hydroelectric nanogenerators: a mini-review. *Cellulose* **30**, 4071-4095 (2023).

12 Youm, J. *et al.* Highly increased hydrovoltaic power generation via surfactant optimization of carbon black solution for cellulose microfiber cylindrical generator. *Surf. Interfaces* **38**, 102853 (2023).

13 Xie, J. H., Wang, Y. F. & Chen, S. G. Textile-based asymmetric hierarchical systems for constant hydrovoltaic electricity generation. *Chem. Eng. J.* **431**, 133236 (2022).

14 Butler, J. M., Johnson, J. E. & Boone, W. R. The heat is on: room temperature affects laboratory equipment--an observational study. *J. Assist. Reprod. Genet.* **30**, 1389-1393 (2013).

15 Sun, Z., Zhang, W., Guo, J., Song, J. & Deng, X. Is heat really beneficial to water evaporation-driven electricity? *J. Phys. Chem. Lett.* **12**, 12370-12375 (2021).

16 Han, Y., Zhang, J., Hu, R. & Xu, D. High-thermopower polarized electrolytes enabled by methylcellulose for low-grade heat harvesting. *Sci. Adv.* **8**, eabl5318 (2022).

17 Wang, Y. *et al.* In situ photocatalytically enhanced thermogalvanic cells for electricity and hydrogen production. *Science* **381**, 291-296 (2023).

18 Persad, A. H. & Ward, C. A. Expressions for the evaporation and condensation coefficients in the Hertz-Knudsen relation. *Chem. Rev.* **116**, 7727-7767 (2016).

19 Marek, R. & Straub, J. Analysis of the evaporation coefficient and the condensation coefficient of water. *Int. J. Heat Mass Tran.* **44**, 39-53 (2001).

20 Barrett, J. & Clement, C. Kinetic evaporation and condensation rates and their coefficients. *J. Colloid Interf. Sci.* **150**, 352-364 (1992).

21 Jin, H. *et al.* Identification of water-infiltration-induced electrical energy generation by ionovoltaic effect in porous CuO nanowire films. *Energy Environ. Sci.* **13**, 3432-3438 (2020).

22 Yun, T. G. *et al.* Ion-permselective conducting polymer-based electrokinetic generators with maximized utility of green water. *Nano Energy* **94**, 106946 (2022).

23 Wang, Z. *et al.* Unipolar solution flow in calcium-organic frameworks for seawater-evaporation-induced electricity generation. *J. Am. Chem. Soc.* **146**, 1690-1700 (2024).

24 Qin, Y. *et al.* Constant electricity generation in nanostructured silicon by evaporation-driven water flow. *Angew. Chem. Int. Ed.* **59**, 10619-10625 (2020).

25 Ma, Q. *et al.* Rational design of MOF-based hybrid nanomaterials for directly harvesting electric energy from water evaporation. *Adv. Mater.* **32**, e2003720 (2020).

26 Yang, P. H. *et al.* Solar-driven simultaneous steam production and electricity generation from salinity. *Energy Environ. Sci.* **10**, 1923-1927 (2017).

27 Fang, S., Lu, H., Chu, W. & Guo, W. Mechanism of water-evaporation-induced electricity beyond streaming potential. *Nano Res. Energy* **3**, e9120108 (2024).

28 Li, P. *et al.* Multistage coupling water-enabled electric generator with customizable

energy output. *Nat. Commun.* **14**, 5702 (2023).

29 Li, L. H. *et al.* A novel, flexible dual-mode power generator adapted for wide dynamic range of the aqueous salinity. *Nano Energy* **85**, 105970 (2021).

30 Shao, C. *et al.* Large-scale production of flexible, high-voltage hydroelectric films based on solid oxides. *ACS Appl. Mater. Interfaces* **11**, 30927-30935 (2019).

31 Liu, X. *et al.* Power generation from ambient humidity using protein nanowires. *Nature* **578**, 550-554 (2020).

32 Fang, S. M., Li, J. D., Xu, Y., Shen, C. & Guo, W. L. Evaporating potential. *Joule* **6**, 690-701 (2022).

33 Li, Z. *et al.* Polyaniline-Coated MOFs Nanorod Arrays for Efficient Evaporation-Driven Electricity Generation and Solar Steam Desalination. *Adv. Sci.* **8**, 2004552 (2021).

34 Hou, B. *et al.* Flexible and portable graphene on carbon cloth as a power generator for electricity generation. *Carbon* **140**, 488-493 (2018).

35 Xiao, P. *et al.* Exploring interface confined water flow and evaporation enables solar-thermal-electro integration towards clean water and electricity harvest via asymmetric functionalization strategy. *Nano Energy* **68** (2020).

36 Hou, B. *et al.* Flexible graphene oxide/mixed cellulose ester films for electricity generation and solar desalination. *Appl. Therm. Eng.* **163**, 114322 (2019).

37 Li, Z., Chen, D., Gao, H., Xie, H. & Yu, W. Reduced graphene oxide composite nanowood for solar-driven interfacial evaporation and electricity generation. *Appl. Therm. Eng.* **223**, 119985 (2023).

38 Ge, C. *et al.* Fibrous solar evaporator with tunable water flow for efficient, self-operating, and sustainable hydroelectricity generation. *Adv. Funct. Mater.* **34**, 2403608 (2024).
