# Supplementary material for: Hermetic hydrovoltaic cell sustained by internal water circulation
Source: Nat Commun. 2024 Nov 12;15:9796. doi: 10.1038/s41467-024-54216-y (PMC11557918; doi:10.1038/s41467-024-54216-y)
Supplement: Supplementary file 2 — Description of Additional Supplementary Files [file 41467_2024_54216_MOESM2_ESM.pdf]

Description of Additional Supplementary Files

File Name: Supplementary Video 1

Description: Electronics powered up by HHC array. A 24-HHC-array that is connected in series can power up commercial electronic devices with capacitors, including LCD screens, digital clocks, calculators and LEDs. A clock is used to display the actual time.
